# MitoSwap – Mitophagy partnered with compensatory mitochondrial biogenesis during stem cell differentiation

**DOI:** 10.1080/27694127.2022.2071549

**Published:** 2022-05-04

**Authors:** Priyanka Gajwani, Jalees Rehman

**Affiliations:** a Department of Pharmacology and Regenerative Medicine, University of Illinois, College of Medicine, Chicago, IL 60612; b Division of Cardiology, Department of Medicine, University of Illinois, College of Medicine, Chicago, IL 60612; c University of Illinois Cancer Center, Chicago, IL 60612

**Keywords:** CTNNB1/β-catenin, differentiation, endothelium, mitochondrial biogenesis, mitofusin 2, mitophagy, PINK1, PPARGC1A/PGC1α, stem cells

## Abstract

Differentiating stem cells must adapt their mitochondrial metabolism to fit the needs of the mature differentiated cell. In a recent study, we observed that during differentiation to an endothelial phenotype, pluripotent stem cell mitochondria are removed by mitophagy, triggering compensatory mitochondrial biogenesis to replenish the mitochondrial pool. We identified the mitochondrial phosphatase PGAM5 as the link between mitophagy and transcription of the mitochondrial biogenesis regulator PPARGC1A/PGC1α in the nucleus. Swapping of mitochondria through the coupled processes of mitophagy and mitochondrial biogenesis lead to enhanced metabolic reprogramming in the differentiated cell.

The mitochondrial metabolism of pluripotent stem cells is characterized by a high dependence on glutamine as a carbon source and thus quite different from the metabolism of differentiating cells which are more attuned to the metabolic needs of their mature cell function. Examples of a mature cell metabolic programming include increased mitochondrial fatty acid oxidation in cardiomyocytes which require large amounts of ATP to enable cardiac contraction or proliferating endothelial cells which use nucleotides generated from fatty acid oxidation. This raises intriguing questions about the shift in mitochondrial metabolism that occurs when pluripotent stem cells differentiate: Are existing stem cell mitochondria remodeled to fit their post-differentiation needs? Or is the pluripotent stem cell mitochondrial pool removed and replaced with mitochondria that are better suited for the mature cell metabolic needs? If the latter, how are mitochondrial removal and replacement coordinated?

Inefficient or damaged mitochondria are removed via mitophagy as part of routine mitochondrial quality control, most notably through the PINK1-MFN2 (mitofusin 2)-PRKN pathway. Typically, stressors such as high oxidative stress damage activate mitophagy but a physiological process such as differentiation might also use mitophagy to selectively remove mitochondria associated with the pluripotent state. We therefore investigated whether mitophagy occurs during the 7-day differentiation of human induced pluripotent stem cells (iPSCs) to a vascular endothelial phenotype [1]. While undergoing endothelial differentiation, iPSCs first transition to a mesodermal progenitor state and then to an endothelial progenitor state. We observed that the cells exhibit a surge of mitophagy during these initial phases of differentiation before a return to baseline levels of mitophagy once fully differentiated. Similarly, we found increased protein levels of PINK1, and increased phosphorylation of MFN2, both indicators of increased mitophagy through the PINK1-PRKN pathway. This mitophagic removal of mitochondria is one indication that during stem cell differentiation into endothelial cells, metabolic reprogramming of mitochondria likely involves wholesale replacement of mitochondria instead of merely “upgrading” existing stem cell mitochondria.

We next examined whether this observed mitophagy during differentiation was coupled to compensatory mitochondrial biogenesis. We measured the production of new mitochondria using the biosensor MitoTimer, a doxycycline-inducible mitochondrially-targeted fluorescent protein which initially displays green fluorescence, but over time converts to a red fluorescent protein. Thus, it is possible to date mitochondria by assessing fluorescence ratios. Compared to pluripotent stem cells, endothelial progenitor cells display a significantly higher ratio of new:old mitochondria. Additionally, we found significantly increased expression of the mitochondrial biogenesis regulator PPARGC1A/PGC1α in the endothelial progenitor state and an increase in mitochondrial mass as the cells transitioned to the differentiated endothelial state, suggesting that the increase in new:old mitochondria is indeed due to *de novo* biogenesis and not merely the result of selectively removing “older” mitochondria through mitophagy.

We next examined whether the observed swapping of mitochondria by the mitophagy-mitochondrial biogenesis coupling generates more ATP. We depleted the mitophagy mediator MFN2 and measured ATP levels in pluripotent stem cells as well as endothelial progenitor cells and found that endothelial progenitors display a significantly higher ratio of ATP:ADP when compared to undifferentiated stem cells. However, this increased ATP is blunted by the deletion of *MFN2*, suggesting that differentiation-associated mitophagy helps promote a metabolic shift towards increased ATP production. How does this shift come about? Compared to iPSCs, differentiated endothelial cells increase the nuclear expression of fatty acid metabolism genes, and decrease the expression of glutamine metabolism genes. Differentiated endothelial cells also showed increased oxygen consumption when exposed to the fatty acid palmitate as a carbon source.

We next wondered how mitophagy and *de novo* mitochondrial biogenesis are coordinated to ensure the seamless replacement of the recycled mitochondria. As the vast majority of mitochondrial proteins are encoded by the nucleus, we surmised that mitophagy likely activates cues which signal to the nucleus that there is a need for *de novo* mitochondrial biogenesis. The mitochondrial phosphatase PGAM5 was recently identified as a driver of nuclear CTNNB1/β-catenin-driven gene expression in HEK293T cells. During mitophagy, PGAM5 is cleaved and released from the mitochondrial membrane, where it can then interact with CTNNB1/β-catenin in the cytosol. As a phosphatase, PGAM5 dephosphorylates CTNNB1/β-catenin, thus preventing its degradation and allowing it to translocate to the nucleus where it acts as a transcription cofactor to drive gene expression. We surmised that mitochondria of differentiating stem cells may also release PGAM5 during mitophagy. We indeed observed PGAM5 cleavage during differentiation as well as increased CTNNB1/β-catenin transcriptional activity in endothelial progenitor cells. Depletion of PGAM5 by siRNA reduces expression of the mitochondrial biogenesis regulator PPARGC1A. Thus, differentiation-induced mitophagy likely communicates with the nucleus through the PGAM5-CTNNB1/β-catenin axis to initiate compensatory biogenesis via PPARGC1A (Figure 1).

**Figure 1.**
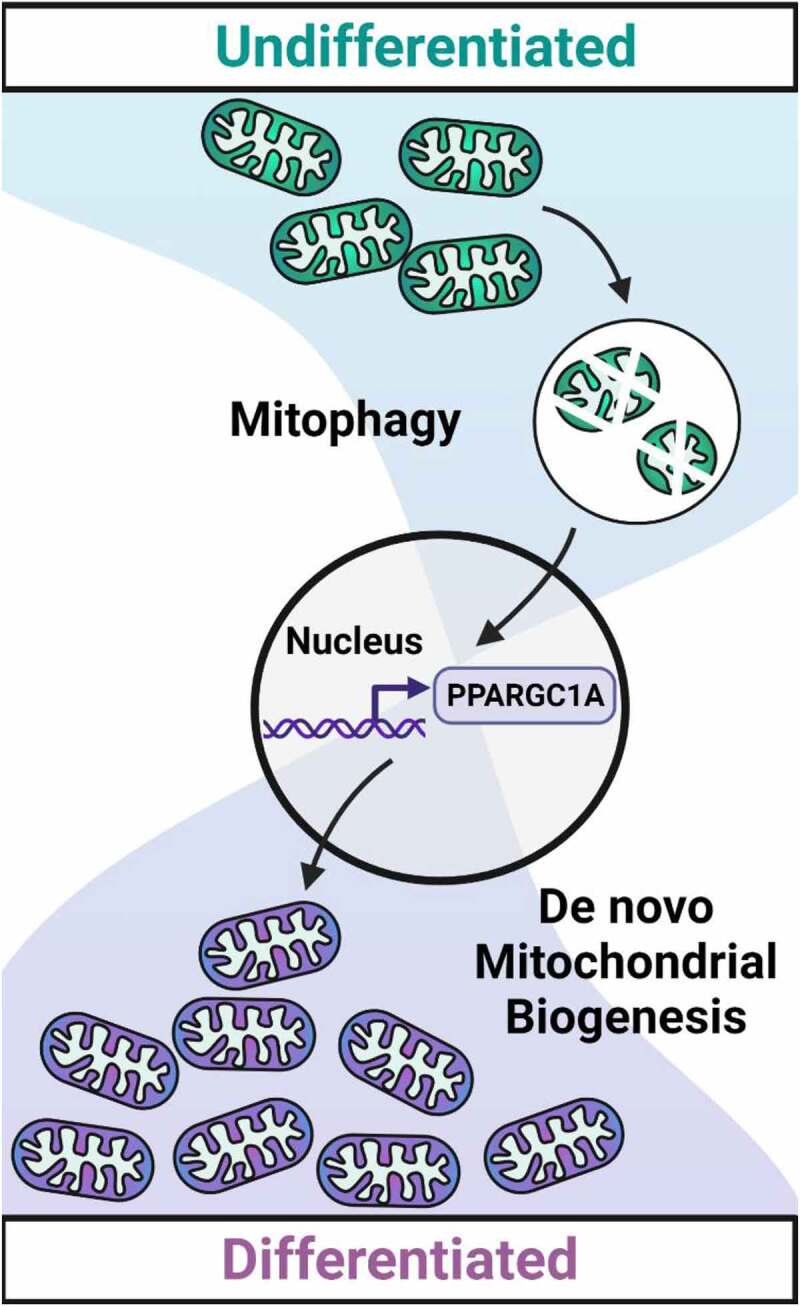
Schematic of differentiation-induced mitochondrial swapping. During differentiation, iPSC mitochondria are degraded and replaced by new mitochondria that are more suited to the mature differentiated cell. Mitochondrial degradation through mitophagy directs the nuclear transcription of mitochondrial biogenesis regulators such as PPARGC1A/PGC1α (PPARG coactivator 1 alpha), coupling the removal and synthesis processes, and enabling efficient metabolic reprogramming. Created with Biorender.com.

There are several intriguing questions that can be addressed in future studies. Is the differentiation-induced mitophagy and its coupling to compensatory mitochondrial biogenesis a universal phenomenon for all forms of stem cell differentiation? Or are there certain cell differentiation trajectories in which existing mitochondria are “upgraded” with newly expressed mitochondrial proteins as a form of metabolic adaptation? Do the differentiation cues direct the metabolic program of the de novo generated mitochondria? For example, how would the growth factors governing cardiomyocyte differentiation, hepatocyte differentiation and neuronal differentiation regulate the expression of mitochondrial enzymes during the replenishment process to optimize the mitochondria for the respective cell functions? In summary, our study outlines the coupling of mitophagy and compensatory mitochondrial reprogramming during the differentiation of stem cells and provides new insights into metabolic reprogramming, raising important broader questions about the role of mitophagy in stem cell differentiation.
